# The EHA Research Roadmap: Platelet Disorders

**DOI:** 10.1097/HS9.0000000000000601

**Published:** 2021-06-30

**Authors:** Carlo Balduini, Kathleen Freson, Andreas Greinacher, Paolo Gresele, Thomas Kühne, Marie Scully, Tamam Bakchoul, Paul Coppo, Tadeja Dovc Drnovsek, Bertrand Godeau, Yves Gruel, A. Koneti Rao, Johanna A. Kremer Hovinga, Michael Makris, Axel Matzdorff, Andrew Mumford, Alessandro Pecci, Hana Raslova, José Rivera, Irene Roberts, Rüdiger E. Scharf, John W. Semple, Christel Van Geet

**Affiliations:** 1 Ferrata-Storti Foundation, Pavia, Italy; 2 Department of Carzdiovascular Sciences, Center for Molecular and Vascular Biology, University of Leuven, Belgium; 3 Institut für Immunologie und Transfusionsmedizin, Universitätsmedizin Greifswald, Germany; 4 Section of Internal and Cardiovascular Medicine, Department of Medicine and Surgery, University of Perugia, Italy; 5 University Children’s Hospital Basel, Oncology/Hematology, Basel, Switzerland; 6 Cardiometabolic Programme, Biomedical Research Center, University College London, United Kingdom; 7 University Hospital of Tübingen, Germany; 8 Service d'Hématologie, Hôpital Saint Antoine, Sorbonne-Université, French Reference Center for Thrombotic Microangiopathies, Centre de Recherche des Cordeliers, Paris, France; 9 Blood Transfusion Centre of Slovenia, Ljubljana, Slovenia; 10 Département de médecine interne, Hôpitaux Universitaires Henri Mondor, Université Paris Est Créteil, Créteil, France; 11 Department of Haematology-Haemostasis, Tours University Hospital, Tours, France; 12 Sol Sherry Thrombosis Research Center and Hematology Section, Lewis Katz School of Medicine at Temple University, Philadelphia, Pennsylvania, United States; 13 Department of Hematology and Central Hematology Laboratory, Inselspital, Bern University Hospital, University of Bern, Switzerland; 14 Royal Hallamshire Hospital, Sheffield, United Kingdom; 15 Department of Internal Medicine II, Asklepios Clinic Uckermark, Schwedt, Germany; 16 School of Cellular and Molecular Medicine, University of Bristol, United Kingdom; 17 Department of Internal Medicine, Fondazione Istituto di Ricerca e Cura a Carattere Scientifico Policlinico San Matteo and University of Pavia, Italy; 18 Institut national de la santé et de la recherche médicale, Unité mixte de recherche 1287, Gustave Roussy, Université Paris Saclay, Equipe labellisée Ligue Nationale contre le Cancer, Villejuif, France; 19 Servicio de Hematología y Oncología Médica, Hospital Universitario Morales Meseguer, Centro Regional de Hemodonación, University of Murcia, Murcia, Spain; 20 Department of Paediatrics and Medical Research Council Molecular Haematology Unit, Medical Research Council Weatherall Institute of Molecular Medicine, Oxford, United Kingdom; 21 Heinrich Heine University Medical Centre, Düsseldorf, Germany; 22 Division of Hematology and Transfusion Medicine, Department of Laboratory Medicine, Lund University, Sweden; 23 Department of Cardiovascular Sciences, KU Leuven, Belgium

*In 2016, the European Hematology Association (EHA) published the EHA Roadmap for European Hematology Research* ^1^ *aiming to highlight achievements in the diagnostics and treatment of blood disorders, and to better inform European policy makers and other stakeholders about the urgent clinical and scientific needs and priorities in the field of hematology. Each section was coordinated by 1 to 2 section editors who were leading international experts in the field. In the 5 years that have followed, advances in the field of hematology have been plentiful. As such, EHA is pleased to present an updated Research Roadmap, now including 11 sections, each of which will be published separately. The updated EHA Research Roadmap identifies the most urgent priorities in hematology research and clinical science, therefore supporting a more informed, focused, and ideally a more funded future for European hematology research. The 11 EHA Research Roadmap sections include Normal Hematopoiesis; Malignant Lymphoid Diseases; Malignant Myeloid Diseases; Anemias and Related Diseases; Platelet Disorders; Blood Coagulation and Hemostatic Disorders; Transfusion Medicine; Infections in Hematology; Hematopoietic Stem Cell Transplantation; CAR-T and Other Cell-based Immune Therapies; and Gene Therapy.*

Since the publication in 2016 of the first EHA consensus document on the Roadmap for European Hematology Research,^1^ many and important advances in the field of platelet disorders have been achieved. Figures 1 and 2, taken from collaborative European studies, exemplify the relevance of these findings to clinical practice. As always happens in scientific research, new knowledge opened up new fields of investigation and identified new objectives to be achieved. Consequently, this Roadmap edition contains numerous new suggestions for studies in the coming years. Scientific progress is indeed a never-ending journey, and the clinicians and basic researchers who contributed to this section, all top scientists in the field of platelet disorders, clearly indicate which directions to go.

**Figure 1.**
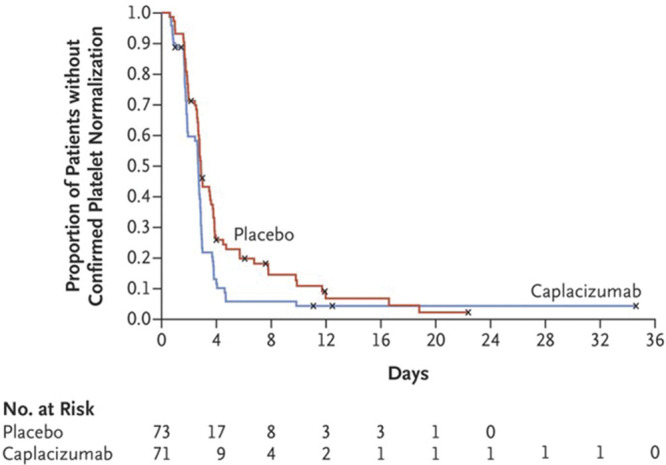
**Caplacizumab improves prognosis of acquired TTP.** This large European study revealed that caplacizumab added to plasma exchange shortens the time for normalization of the platelet count. This translates in a lower incidence of a composite of TTP-related death, recurrence of TTP, a thromboembolic event during the treatment period, and a lower rate of recurrence of TTP.^2^ Copyright ^©^ 2019 Massachusetts Medical Society. Reprinted with permission from Massachusetts Medical Society. TTP = thrombotic thrombocytopenic purpura.

**Figure 2.**
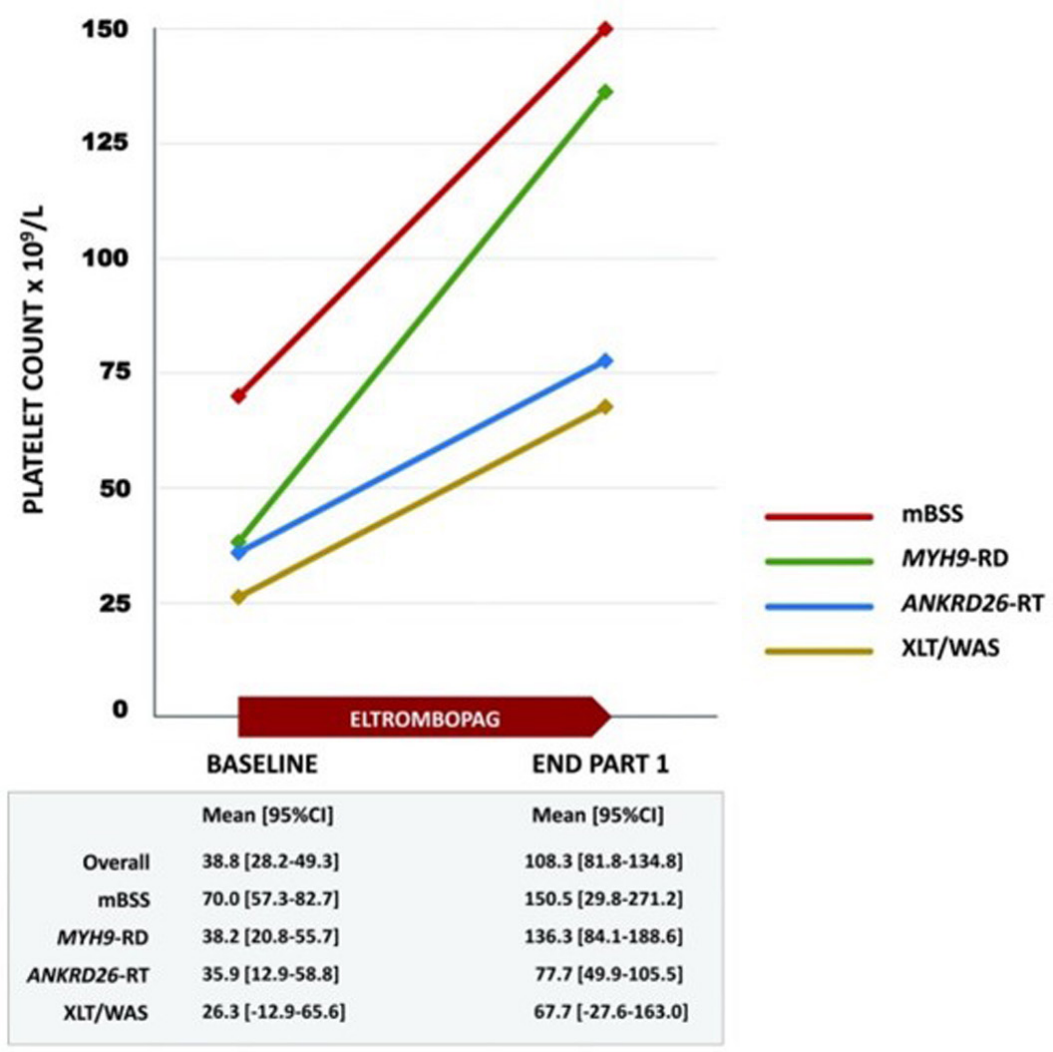
**Eltrombopag increases platelet count in inherited thrombocytopenias.** In this collaborative study, the thrombopoietin-receptor agonist eltrombopag brought the number of platelets to a safe level in patients with 4 different forms of inherited thrombocytopenia. The authors concluded that this drug can be used in place of platelet transfusions to prepare patients for hemostatic challenges. Reprinted with permission from Zaninetti et al.^3^ ANKRD26-RT = ANKRD26-related thrombocytopenia; mBSS = monoallelic Bernard-Soulier syndrome; MYH9-RD = MYH9-related disease; XLT/WAS = X-linked thrombocytopenia/Wiskott-Aldrich syndrome.

The challenge to achieve these objectives is not only scientific, but also economic. Research is in fact increasingly expensive and obtaining the required funding is difficult, especially in the field of rare diseases to which many of the platelet disorders belong. The challenge is also organizational, as it is clear that research, especially clinical research, on rare diseases is all the more productive and fast the more research groups and countries are involved. This idea is shared by all the authors of this section, who in fact attribute many of the most important recent successes to large collaborative studies and indicate collaboration as one of the essential tools for further improving care of subjects with platelet disorders. Despite progress in this direction has recently been made, some forms of thrombocytopenia still suffer from poor attention and a lack of dedicated resources (Table 1).

**Table 1.** Clinical Trial on Thrombocytopenias and Platelet Function Disorders that Have Been Registered in the European Clinical Trials Register Over the Past 5 y.

Platelet Disorder or Group of Disorders	Number of Registered Trials
Immune thrombocytopenia	18
Thrombotic microangiopathies	12
Heparin-induced thrombocytopenia	1
Fetal neonatal alloimmune thrombocytopenia	1
Inherited thrombocytopenias	1
Inherited or acquired disorders of platelet function	0

It is clear that there is a serious lack of clinical studies for some forms of platelet disorders, both rare and frequent.

## Congenital platelet disorders: number and function

Kathleen Freson, Paolo Gresele, Andrew Mumford, Alessandro Pecci, Hana Raslova, José Rivera

Congenital platelet disorders (CPD) are caused by germline variation in genes involved in the production and/or function of platelets.^4^ CPD are extremely heterogeneous, with over 60 genes now known to be associated.^5^ Clinical and laboratory investigation of patients phenotypes has always played a central role in the diagnostics of CPD for a long time,^6^ with Sanger sequencing only performed in genes where a defect was suspected. During the last decade, several next generation sequencing (NGS) approaches have been used to study CPD, resulting in major advances in this field.

## European research contributions

Different groups in Europe have contributed to the discovery of more than 20 novel genes using whole exome or whole genome sequencing (WES/WGS) but for many of these genes, their exact function in platelets and/or megakaryocytes still remains unknown.^4,5^ In parallel, diverse clinical laboratories have implemented NGS techniques to survey multiple genes simultaneously for diagnostic purposes.^7^ A conclusive diagnosis using NGS tests can be obtained quickly and cost-effectively in a significant proportion of patients, with diagnostic rates varying between 26.1% and 46.8% for platelet function and number disorders, respectively.^8^ The expanding use of NGS tests has raised concerns regarding complex variant interpretation and the ethical implications of detecting unsolicited findings such as variants in RUNX1, ETV6, and ANKRD26, which are associated with increased leukemic risk.^9,10^ Guidelines for consenting and variant interpretation are critical for safe patient care. Efforts have been undertaken to develop in vitro models of platelet biogenesis^11^ as powerful tools to validate the pathogenicity of variations identified by NGS.

## Proposed research for the roadmap

To implement multigene panel testing using NGS approaches (targeted, WES or WGS) for CPD: single-step sequencing of “diagnostic-grade” genes as the first-line diagnostic approach for patients with CPD may prove more time and cost-effective than traditional Sanger sequencing. The following steps are encouraged: development of guidelines related to informed consent documents and opt_in/opt_out possibilities for leukemia risk genes; to stimulate reporting variants in open access variant databases for improved variant classification; development of easy-to-use variant selection platforms; introduction of WGS in frontline clinical diagnostics.

To establish registries of CPD patients with known pathogenic variants: systematic data collection supported by Findable, Accessible, Interoperable and Reusable (FAIR) data practices, to define the clinical consequences of pathogenic variants through the systematic investigation of large patient cohorts (including follow-up of clonal hematopoiesis in patients with mutations in RUNX1, ETV6, and ANKRD26). Moreover, to carry out a worldwide, multicentric, prospective study to assess the bleeding complications of surgery; to promote systematic surveys on the efficacy of prophylactic platelet transfusions before surgery or delivery and develop guidelines; to investigate modulating factors of clinical severity other than the causative pathogenic variants.

To identify new genes responsible for unexplained CPD: supplementation of WES or WGS datasets of unexplained patients with platelet RNA-sequencing and proteomics data; to stimulate gene discovery programs on specific platelet disorder groups that still have very low diagnostic yields; to promote additional case reports with variants in recently discovered genes for CPD that still require confirmation.

To use cell-based models to study platelet formation and function for disease modeling as well as preclinical studies of novel therapies: to promote the use of disease models based on patient-derived hematopoietic stem cells and inducible pluripotent stem cells. To improve these models in order to reproduce more closely the bone marrow microenvironment for the study of platelet production and the conditions of human circulation to study platelet functional defects.

To explore novel therapeutic options for CPD: focus on thrombopoietin mimetics and drugs that stimulate megakaryopoiesis with a thrombopoietin-independent mechanism for the treatment of thrombocytopenia and gene therapy studies for the most severe forms of CPD.

## Anticipated impact of the research

Defining the clinical phenotypes derived from pathogenic variants is the basis for providing CPD patients with a personalized, genotype-driven prognostic assessment, and therefore to set the appropriate follow-up, choose the best treatments and offer correct genetic counseling.

## Acquired nonimmune thrombocytopenia and acquired disorders of platelet function

Paolo Gresele, Michael Makris, A Koneti Rao, Rüdiger E. Scharf, Christel Van Geet

Acquired nonimmune disorders of platelet function and number (APD), different from immune thrombocytopenia (ITP), are very common but relatively poorly studied. Chronic kidney or liver diseases are associated with platelet dysfunction but their clinical relevance is unclear. Many drugs and food transiently modify platelet function and may associate with increased bleeding. Cardiovascular procedures involving blood exposure to foreign surfaces, like cardiopulmonary bypass (CPB), left ventricular assist devices (LVAD), and extracorporeal membrane oxygenation (ECMO) induce APD. Finally, nonimmune thrombocytopenia (NITP) is frequent in acutely ill patients, especially with infection. However, this condition is not well understood.^12^

## European research contributions

European researchers have strongly contributed to characterize APD in chronic liver and kidney disease.^13–15^ The bleeding risk of drug-induced platelet dysfunction in patients undergoing surgery and its management^15^ as well as the platelet dysfunction of LVAD and its role in bleeding complications have become better understood.^16^ European studies have documented the predictive value of thrombocytopenia for adverse outcome in critically ill patients and have described mechanisms causing thrombocytopenia.^17,18^

## Proposed research for the roadmap

To clarify the clinical relevance of APD in chronic liver and kidney disease by a large international, prospective study on the prognostic value of the International Society for Thrombosis and Haemostasis bleeding score. Studies employing novel and sensitive techniques for the assessment of platelet/vessel wall interactions and correlation with clinical bleeding are warranted. A rational diagnostic algorithm for the identification of clinically relevant APD and its best management needs to be established by prospective studies. The role of platelets in liver regeneration and fibrosis and its possible pharmacologic modulation require exploration.

To define the clinical relevance and best management of drug-induced platelet dysfunction in patients undergoing surgery/invasive procedures, by a large retrospective survey assessing the relationship between presurgical drug-intake and surgical bleeding.^19^ Organ transplantation-induced APD and the potential influence of drugs on post-transplantation bleeding should be assessed. The possibility to guide surgery in antiplatelet-treated patients by preoperative platelet function testing requires evaluation.

To unravel the mechanisms and improve diagnosis and treatment of infection-associated thrombocytopenia (IATP). Differentiation of IATP from ITP and other acquired thrombocytopenias is crucial because treatment is different. Issues to be addressed are whether platelet count profiles differ depending on the pathogen; if the immature platelet fraction helps in diagnosis; what is the bleeding risk of IATP; if thrombopoietin (TPO)-agonists are efficacious; what is the expression profile of cell-derived microparticles; what platelet-induced inflammatory responses can be protective or detrimental. To address these issues, a IATP registry, prospective multicenter studies and murine sepsis models will have to be established. Finally, there is an urgent need to understand the mechanisms of platelet activation and consumption in coronavirus disease 2019 (COVID-19) and the platelet receptors involved.^20,21^

To understand the contribution of APD to the bleeding complications of CPB, LVAD and ECMO. Exposure of flowing blood and abnormal shear-stress within the extracorporeal circuit are major determinants of hemostatic derangements. Impaired platelet adhesion, activation, and aggregation have been shown^22^ and may cause otherwise unexplained bleeding.^23–25^ Well-designed studies characterizing the platelet dysfunction and its mechanism during CPB, LVAD, and ECMO are required.^26^

To differentiate ITP from NITP. The clear and simple differentiation between ITP and acquired NITP is a crucial task of future research. In particular, better and more rapid tests for the confirmation of ITP and a European collaborative network for thrombocytopenia in pregnancy, to understand its natural history in mothers and fetuses, are required.

To comprehend APD in myeloproliferative neoplasms (MPN) in relation to gene-mutation and drug treatments, by promoting large, prospective collaborative studies in MPN patients correlating platelet function parameters with JAK-2, CALR, or MPL mutations and type of treatment (JAK-inhibitors, histone deacetylase-inhibitors, telomerase-inhibitors and human double minute 2-inhibitors).

## Anticipated impact of the research

The clarification of the clinical meaning of APD in disorders of large epidemiological impact may provide a guide to their diagnosis and management. The understanding of the impact of drug-induced platelet dysfunction on surgical bleeding and its possible prediction by laboratory testing may reduce morbidity and mortality. The proposed research will provide further insight into the mechanisms of thrombocytopenia associated with infections, including COVID-19, and in their management. Finally, the differentiation between ITP and NITP will allow more appropriate and rapid treatment.

## Primary and secondary immune thrombocytopenia and fetal neonatal alloimmune thrombocytopenia

Thomas Kühne, Bertrand Godeau, Axel Matzdorff, Irene Roberts, John W. Semple

ITP is a rare bleeding disorder and qualifies as an orphan disease by the definition of the European Medicines Agency. It is an acquired autoimmune disorder characterized by isolated thrombocytopenia due to pathogenic antiplatelet autoantibodies, T cell–mediated platelet destruction, and impaired megakaryocyte function. Autoantibody-opsonized platelets are recognized by Fcγ-receptor positive macrophages that results in enhanced platelet phagocytosis and destruction in the spleen. Autoantibodies may inhibit megakaryocyte maturation and their destruction. Autoreactive T cells are also involved in both platelet and megakaryocyte destruction. Subsets of these lymphocytes have become known to be indispensable for platelet autoimmunity and drive the autoimmune response.

Although treatment of patients with ITP has improved, it is paradoxically more complex and most countries in Europe produced practice guidelines.

Fetal and neonatal alloimmune thrombocytopenia (FNAIT) is also rare, due to fetomaternal alloimmunization to paternal human platelet antigens (HPA). However, it is one of the commonest causes of severe thrombocytopenia in otherwise healthy neonates and carries with it a high risk of hemorrhage-associated mortality and long-term disability. It has become clear that noninvasive management is as effective as fetal blood sampling-based approaches. Weekly maternal administration of immunoglobulins is the most appropriate first-line antenatal treatment of FNAIT with no consistent evidence for adding steroids.

## European research contributions

For ITP and FNAIT, European researchers are playing a key role in epidemiology, clinical and basic research. Systematic reviews have crystallized the evidence for recommendations about the antenatal management of FNAIT. European groups have led the way in developing, testing, and implementing screening programs for FNAIT. The rarity of ITP demands collaborative research. Several European initiatives evolved in basic and clinical research including the European Research Consortium on ITP as well as national and international registries.

## Proposed research for the roadmap

Pathophysiology of ITP. Understanding the immune pathophysiology of ITP is critical to developing novel and targeted therapies. A challenge represents the identification of a biomarker of ITP so that an accurate test can be developed. The study of the autoimmune mechanisms from patients without autoantibodies (20%–40%) is a research priority.^27,28^ It is still unknown how autoreactive T cells mediate ITP, but nonetheless, they are necessary for autoimmune progression. They may exhibit enhanced resistance to apoptosis and elevated clonal expansion rates. The study of the regulation and maintenance of immune tolerance and of megakaryo- and thrombopoiesis is also a research priority.

Pathophysiology and management of FNAIT. Defining the role of screening for FNAIT remains a key question. The development of new diagnostic tools is essential for improving management. Moreover, the study of ante- and postnatal management is important and includes the prospective investigation of risk stratification.^29^ Prophylactic approaches, such as a hyperimmune anti-HPA-1a immunoglobulin to prevent anti-HPA-1a formation will take many years.^30^ The impact on clinical outcome of FNAIT of more recently discovered antibody characteristics such as fucosylation and glycosylation patterns, as well as MHC allele associations will require collaborative initiatives. The prognostic value of HPA antibody levels should also be investigated.

Clinical proposals in children and adults with ITP. Pediatric chronic symptomatic ITP should be studied with development of diagnostic algorithms. The management of ITP with treatment endpoints other than the platelet count is currently undergoing fundamental changes and treatment algorithms have to be adapted to these developments.^31^ Chronic ITP affects many aspects of patients’ quality of life with measurable treatment outcomes, such as bleeding, quality of life, and fatigue.^32,33^ The early detection of chronic ITP patients is of clinical and economic value.^34^

New treatments of ITP. First-line therapies are undergoing new approaches, such as early treatment intensification, combined treatment approaches, and upfront use of TPO receptor agonists en lieu or combined with corticosteroids to restore an immune tolerance and induce remission in a greater proportion compared with a corticosteroid-monotherapy.^35^ The study of second-line therapies include efficacy, safety, combinations of drugs (particularly rituximab), and comparisons of known and new drugs (avatrombopag, lusutrombopag, Bruton tyrosine kinase, neonatal Fc receptor-, complement, and spleen associated tyrosine kinase inhibitors).^36^

Finally, new algorithms are needed to take into account the European economic constraints, quality of life, and the opinion of patients.

## Anticipated impact of the research

The systematic study of immune pathways will provide more insights into FNAIT and ITP and may identify new diagnostic tools and algorithms to better define patient prognosis and more personalized therapeutic approaches.

## Heparin-induced thrombocytopenia and other drug-dependent immune thrombocytopenias

Andreas Greinacher, Tamam Bakchoul, Yves Gruel, Tadeja Dovc Drnovsek

Drug-induced ITPs (DITPs) including heparin-induced thrombocytopenia (HIT), result from drug-dependent antibodies (DDABs), inducing platelet destruction or activation. DITPs are (1) life threatening and require rapid recognition to allow appropriate measures to avoid harm; (2) relevant for drug approval, with a major economic impact on the development of new compounds. The most frequent DITP is HIT,^37^ which is currently the underlying cause of >95% of all confirmed DITPs. While most DITPs increase the risk for bleeding, HIT is prothrombotic, and its incidence is 1:10,000 in-hospital patients making DITP a substantial health issue in Europe. A major challenge is the introduction of new biological drugs and genetically engineered cells. Frequency and type of immune reactions toward these new pharmaceutical products are currently largely unknown. This argument became very topical in April 2021 with the demonstration that a vaccine against severe acute respiratory syndrome coronavirus 2 using an adenoviral vector can in rare cases induce a new and serious disease called vaccine-induced immune thrombotic thrombocytopenia (VITT).^38^

Diagnosis of DITPs is based on rather unspecific clinical criteria, and requires confirmation of DDABs by laboratory tests. However, *laboratory tests for non-HIT DITPs* show low sensitivity (but high specificity), are restricted to specialized laboratories and poorly standardized.^39^ In contrast, the widely available *HIT laboratory tests*,^40^ show high sensitivity, but unsatisfactory specificity. The much more specific functional assays are technically demanding and not widely available.

Open issues: strong need for sensitive but also specific screening tests. Access to appropriate testing throughout Europe. Better understanding of the pathogenesis to develop preventive measures. Current treatments of HIT and its clinical sequelae are a major cost burden for hospitals.

## European research contributions

Several European groups made major contributions to the pathogenesis of DITPs,^41^ including the recently discovered VITT,^38^ developed test systems and treatment recommendations. Access of physicians and patients to appropriate laboratory testing is well developed in a number of European countries (France, Germany, Austria, Switzerland, Netherland, United Kingdom). A prospective randomized trial for optimal treatment in HIT is currently underway in several European countries.

## Proposed research for the roadmap

Better understanding the pathogenesis of DITPs: important for drug development, especially new biological drugs. Recent data make it highly likely that platelet factor 4, the main protein in HIT, is involved in pathogen host defense. It acts as a danger-label for the immune system. Further understanding of these mechanisms bears the possibility to optimize antibacteria and antiviral treatments.

Improvement in diagnostic methods of DITPs. Widely applicable assays for DDABs are needed. Especially for HIT, an easy to apply assay with a high positive predictive value is one of the main needs in most laboratories. Establishing networks in Europe providing rapid access to diagnostic assays for DITPs with locally available screening tests with a rapid turnaround time, followed by confirmatory tests with high specificity will be a solution for a currently unmet need.

## Anticipated impact of the research

Adverse immune reactions are the biggest threat for the development of new biotherapeutic drugs. Understanding the underlying mechanisms will not only improve patient safety, but will strengthen European biopharmaceutical industry. DITPs are caused by antibodies reacting with endogenous (self) cells. Identifying the mechanisms at the molecular level will help to understand mechanisms of autoimmunity, as well as misdirected antibacterial or antiviral immune responses.

## Thrombotic thrombocytopenic purpura and other thrombotic microangiopathies

Marie Scully, Paul Coppo, Johanna A. Kremer Hovinga

Thrombotic thrombocytopenic purpura is one of the thrombotic microangiopathies which also includes hemolytic uremic syndrome (HUS) and thrombotic microangiopathies associated with specific predisposing factors. Thrombotic thrombocytopenic purpura (TTP) is due to a severe deficiency in the metalloproteinase, ADAMTS13, whereas HUS not caused by an infective agent, is the result of complement dysregulation.

## European research contributions

The greatest influence in TTP research since 2016 is the completion of 2 randomized controlled trials and licensing of caplacuzimab for the treatment of acute immune-mediated TTP (iTTP).^2,42^ Caplacizumab is now standard of care for patients presenting with acute iTTP, resulting in faster time to platelet count normalization, reduced hospital stay and refractory disease and exacerbations are uncommon occurrences. Moreover, preemptive therapies based on B-cell depletion were shown to protect virtually all patients from clinical relapse.^43–45^ Comparably, in HUS, the completion and licensing of a long-acting complement inhibitor therapy (Ravulizumab), opens up ease of therapy for patients at presentation of HUS but also those requiring long-term therapy, receiving treatment every 8 weeks.^46^

From the previous proposal, an increase in awareness in thrombotic microangiopathies (TMAs) and international collaboration have been the forefront of this progress. Presentations of TTP diagnosis and treatment have continued in regional, national, and international meetings, involving hematology and other disciplines. National reference centers for advice and ADAMTS13 as well as complement testing are established and apart from ADAMTS13 assays, scoring systems, specifically the French scoring system^47^ and the PLASMIC score^48^ are utilized to aid bed-side differentiation of TTP from other forms of thrombotic microangiopathies, while awaiting ADAMTS13 test results. Use of assays involving ADAMTS13 to predict prognosis, outcome,^49^ and need for further therapy^50^ have been successful. Dynamic collaborations between large European registries allow answering specific questions. This collaboration has been expanded by an international terminology paper,^51^ necessary for definitions before proceeding to clinical studies, national TTP/TMA working groups and an international TMA meeting yearly at the American Society of Haematology.

Congenital TTP, until now, has had limited publications involving few cases; unsurprising given the incidence of <1/million/population. However, 2 prominent publications have presented the importance of long-term prophylactic treatment and the risk of end-organ damage despite often normal routine blood counts.^52,53^

For HUS, the international registry group continues to encourage participation of patients and have published important results from specific research questions. As an ultra-rare condition, consensus documentation is often the initial basis for future research, but lead at a European level.^54–58^

## Proposed research for the roadmap and anticipated impact of the research

Use of recombinant ADAMTS 13 in iTTP and congenital TTP (cTTP). Completion of both ongoing studies is critical to future therapy in both types of TTP. For iTTP, the aim will be to avoid plasma exchange. For cTTP, it will ensure ADAMTS13 levels such that subacute microvascular thrombotic events are prevented, precluding early end organ failure.

Presenting a therapeutic, noninvasive pathway for the treatment of acute TTP, involving caplacizumab, recombinant ADAMTS13, and anti-CD20 therapy. Ideally, this should be part of a clinical trial program.

Understanding the longer-term impact of TTP-cognitive symptoms and the role of ADAMTS 13 levels and to what level we should be aiming for in both iTTP and cTTP.

To get further insights in specific subsets of iTTP (in the setting of pregnancy and childhood, in the elderly) with poor level of evidence.

To address the diagnostic and therapeutic unmet needs of thrombotic microangiopathies associated with specific conditions including bone marrow transplantation, cancer and chemotherapy, and systemic autoimmune diseases, where prognosis remains dismal.

Ensuring ongoing collaborative clinical and scientific research between European groups and international colleagues, including countries that to now have been poorly represented.

**Summary box: Main research & policy priorities**

- To perform clinical studies for improving diagnostic and therapeutic strategies for inherited platelet disorders.
- To define the clinical relevance and best management of acquired nonimmune thrombocytopenias and acquired disorders of platelet function.
- To develop new tools to define prognosis and personalize treatment of patients with immune-mediated forms of thrombocytopenia.
- To better understand the pathogenesis of drug-induced immune thrombocytopenias and develop simple diagnostic methods.
- To optimize the therapeutic approach to thrombotic thrombocytopenic purpura with particular attention to secondary forms with poor prognosis.

## Disclosures

KF received research grant from Swedish Orphan Biovitrum (SOBI), royalties payments from Amgen. AG received grants from Deutsche Forschungsgemeinschaft, Ergomed, Boehringer Ingelheim, Rovi, Sagent, Macopharma, Portola, Biokit, Fa. Blau Farmaceutics, Prosensa/Biomarin, DRK-BSD NSTOB, DRK-BSD Baden-Würtemberg/Hessen consultation for Aspen, Chromatec, Instrumentation Laboratory, Macopharma GTH e.V. speakers fee from Bayer Vital, Sanofi-Aventis, Roche. TK is the advisory board member for Bayer, SOBI, Novo. MS received grants from Shire, Novartis, Alexion, speakers fees from Takeda, Alexion, Octapharma, Sanofi, Novartis, advisory board member for Takeda, Sanofi. TB received grants from German Research Foundation (DFG), Robert Bosch Power Tools, German Red Cross gmBH, royalties payments from Aspen Germany GgmbH, Bayer AG, CSL Behring, gGmbH, GSK gGmbH, Stago gGmbH. PC received grants from Sanofi, Alexion, Roche, Octapharma, Janssen, Sandoz; is the advisory board member for Sanofi, Alexion, Takeda, Octapharma; received royalties payments from Sanofi, Alexion, Takeda. BG received royalty payments from Amgen, Novartis, Roche, Grifols, Sobi. AKR received research Grants from the National Institutes of Health (NHLBI). JKH received grants from Bayer, CSL Behring, NovoNordisk, Octapharma, Roche, Sobi, Baxter/Takeda, consultation for Shire/Takeda; Ablynx/Sanofi, lectures fees from Roche, Sanofi, Shire, Takeda, Sobi, Bayer, NovoNordisk, Octapharma, Shire, Sobi, Roche. AM received consultation and royalties payments from Novartis, Amgen, Swedish Orhpan Biovitrium, UCB, Grifols, Roche. A. Mumford received grants from National Institute of Health (US), National Institute of Healthcare Research (UK), British Heart Foundation (UK), Wellcome trust (UK). AP received grants from Telethon Foundation and the IRCCS Policlinico San Matteo Foundation. HR received grants from Ligue Nationale Contre le Cancer, H2020-FETOPEN-1-2016-2017-SilkFusion, Fondation pour la Recherche Medicale. CvG received grants from Bayer, CLS. All the other authors have no conflicts of interest to disclose.
